# Aminolipids elicit functional trade-offs between competitiveness and bacteriophage attachment in *Ruegeria pomeroyi*

**DOI:** 10.1038/s41396-022-01346-0

**Published:** 2022-12-07

**Authors:** Rachel Stirrup, Michaela A. Mausz, Eleonora Silvano, Andrew Murphy, Richard Guillonneau, Mussa Quareshy, Branko Rihtman, Maria Aguilo Ferretjans, Ruo He, Jonathan D. Todd, Feng Chen, David J. Scanlan, Yin Chen

**Affiliations:** 1 School of Life Sciences, University of Warwick, Coventry, CV4 7AL UK; 2 School of Environmental Science and Engineering, Zhejiang Gongshang University, Hangzhou, 310012 China; 3 School of Biological Sciences, University of East Anglia, Norwich Research Park, Norwich, NR4 7TJ UK; 4 Institute of Marine and Environmental Technology, University of Maryland Center for Environmental Science, 701 E. Pratt Street, Baltimore, MD 21202 USA

**Keywords:** Ecology, Microbiology

## Abstract

Lipids play a crucial role in maintaining cell integrity and homeostasis with the surrounding environment. Cosmopolitan marine roseobacter clade (MRC) and SAR11 clade bacteria are unique in that, in addition to glycerophospholipids, they also produce an array of amino acid-containing lipids that are conjugated with beta-hydroxy fatty acids through an amide bond. Two of these aminolipids, the ornithine aminolipid (OL) and the glutamine aminolipid (QL), are synthesized using the *O*-acetyltransferase OlsA. Here, we demonstrate that OL and QL are present in both the inner and outer membranes of the Gram-negative MRC bacterium *Ruegeria pomeroyi* DSS-3. In an *olsA* mutant, loss of these aminolipids is compensated by a concurrent increase in glycerophospholipids. The inability to produce aminolipids caused significant changes in the membrane proteome, with the membrane being less permeable and key nutrient transporters being downregulated while proteins involved in the membrane stress response were upregulated. Indeed, the import of ^14^C-labelled choline and dimethylsulfoniopropionate, as a proxy for the transport of key marine nutrients across membranes, was significantly impaired in the *olsA* mutant. Moreover, the *olsA* mutant was significantly less competitive than the wild type (WT) being unable to compete with the WT strain in co-culture. However, the *olsA* mutant unable to synthesize these aminolipids is less susceptible to phage attachment. Together, these data reveal a critical role for aminolipids in the ecophysiology of this important clade of marine bacteria and a trade-off between growth and avoidance of bacteriophage attachment.

## Introduction

Membrane lipids form the structural basis of a cell separating intracellular compartments and cellular components from the external environment. Glycerol forms the backbone for the synthesis of glycerophospholipids in bacteria, archaea and eukaryotes and much of our understanding of membrane lipids, including the synthesis, biochemistry and biophysics of the lipid bilayer, comes from extensive study in a few model organisms (e.g*., Escherichia coli*). It has long been argued that evolution selected for the use of glycerophospholipids in the membrane of the last common ancestor in the early evolution of cells and indeed, glycerophospholipids are ubiquitous in all forms of life [1, 2].

An important yet severely understudied group of lipids are amino acid-containing aminolipids. Recent work has demonstrated that aminolipids are particularly abundant in ecologically important cosmopolitan marine heterotrophic bacteria. Using the marine roseobacter clade (MRC) bacteria as a model, we have previously demonstrated that the ornithine-containing (OL) and glutamine-containing (QL) aminolipids are common in this bacterial group [3, 4]. Biosynthesis of these lipids is carried out by two distinct enzymes, an *N*-acetyltransferase responsible for the initial conjugation of the corresponding amino acid (i.e., glutamine or ornithine) to a beta hydroxy fatty acid and an *O*-acetyltransferase responsible for the conjugation of a second fatty acid to the lysolipid. In the model marine roseobacter clade bacterium *Ruegeria pomeroyi* DSS-3 OL and QL biosynthesis is mediated by OlsB and GlsB followed by *O*-acetylation by the OlsA protein [4]. OL biosynthesis in some bacteria such as *Vibrio* and *Serratia* can also be mediated by a fusion protein named OlsF which contains an *N*-terminus OlsB-like domain and a *C*-terminus OlsA-like domain [5, 6]. Several other aminolipids have been documented recently, including glycine- and lysine-containing aminolipids in *Bacteroidetes* and *Pseudopedobacter* and a sulfonate-containing aminolipid in roseobacters, respectively [7–9].

The physiological role of these amino acid-containing lipids remains largely uncharacterized. We and others have shown that aminolipids such as OL and QL are common in a range of marine bacteria and are synthesized as surrogate lipids to replace glycerophospholipids in response to phosphorus (P) limitation although in MRC, but not SAR11 strains, these aminolipids are constitutively expressed [4, 10–13]. Indeed, many bacteria produce OL constitutively, regardless of the available P concentration [14]. OL appears to affect the amount of cytochromes in membranes of *Rhodobacter capsulatus* [11]. In *Sinorhizobium meliloti*, OL is overproduced in response to phosphorus stress although it does not play a role in nodulation [12]. Recently, it has been demonstrated that overproduction of OL increases the resistance of *Pseudomonas aeruginosa* to cationic antimicrobials [15]. The role of QL is less well understood. QL appears to be restricted to the marine roseobacter clade and closely related bacteria in the *Rhodobacteraceae* family and appears interchangeable with OL [4].

In this study, we set out to investigate the location of aminolipids and their physiological role in the membrane *of Ruegeria pomeroyi* DSS-3. Using comparative lipidomics and proteomics, we also sought to understand how loss of the OL and QL aminolipids affected inner and outer membrane lipid and protein profiles and membrane transport capacity, by comparing the wild type (WT) strain with an *olsA* mutant deficient in both OL and QL [4]. We demonstrate a critical role for these aminolipids in the membrane of this bacterium with the *olsA* mutant displaying a considerable defect in the abundance and functionality of membrane transporters required for its optimum growth. However, there appears to be a trade-off whereby the aminolipid-deficient strain is less susceptible to phage attachment compared to the wild type. Thus, the bacterium appears to finely balance the production of these lipids in a trade-off between optimum growth and avoiding phage attachment.

## Materials and methods

### Bacterial strains and cultivation

All marine bacteria used in this study were cultivated using the ½ YTSS (yeast-tryptone-sea salt) medium (DSMZ 974), containing yeast extract 2 g/L, tryptone 1.25 g/L and Sigma sea salts 20 g/L or the defined marine ammonium mineral salt (MAMS) medium (DSMZ 1313) where HEPES (10 mM, pH 8.0) replaced the phosphate buffer [16]. All cultures were grown at 30 °C aerobically in a shaker (150 rpm).

For growth competition assays between the WT and the *olsA* mutant, cultures of bacteria were grown in 10 mL ½ YTSS medium for the WT strain, or with the addition of 10 µg/mL gentamicin for the *olsA* mutant since a gentamicin cassette was inserted to construct the mutant [4]. Cells were harvested at mid-late exponential phase and diluted to an optical density measured at 540 nm (OD_540_) of 1.0. These cells were then both inoculated at 1% (v/v) into 250 mL flasks containing 50 mL growth media (either ½ YTSS or MAMS + 0.5 mM Pi) in triplicate and grown at 30 °C with shaking at 140 rpm. At time point 0 h, 100 µL samples were removed in triplicate from each flask. These samples were then ten-fold serially diluted in the same growth media to a dilution of 10^−9^. From each serial dilution tube, 10 µL droplets were pipetted in triplicate onto agar plates containing either ½ YTSS agar (to count both the WT and the *olsA* mutant) or ½ YTSS agar + 10 µg/mL gentamicin (to count just the *olsA* mutant). Once the droplets were dry, plates were incubated at 30 °C for 3-4 days. Colony forming units (CFU) were determined by counting the number of colonies in the dilution number where single colonies were clearly visible. For the cultures grown in ½ YTSS medium, samples were removed and enumerated using the same method at time points 24 h and 96 h. For the cultures grown in MAMS media + 0.5 mM Pi, samples were removed and enumerated at time points 0 h, 48 h and 96 h.

### Membrane separation by sucrose density gradient ultracentrifugation

The WT strain and the *olsA* mutant were grown in ½ YTSS medium to OD_540_ ~0.8. One litre of culture was then collected by centrifugation at 12,300 × *g* at 4 °C for 10 minutes, using a JLA 10.5 rotor. Cells were washed and resuspended in 50 mL HEPES buffer (pH 8.0, 10 mM). Cells were then pelleted by centrifugation at 4,500 × *g* at 4 °C for 10 min, before resuspending the pellet in 3 mL HEPES buffer (pH 8.0, 10 mM), containing 1.6X cOmplete Protease Inhibitor cocktail (Roche), 3X DNAse I buffer (NEB) and 6 units/mL DNase I (NEB). Cells were then lysed using a French Press at 1000 PSI. Cell debris was removed by centrifugation at 4,500 × *g* at 4 °C for 10 min and the supernatant was transferred to a new Oakridge centrifuge tube for pelleting total membranes by centrifugation at 75,600 × *g* at 10 °C for 45 min in a JA25.5 rotor. Pelleted membranes were then washed and resuspended in 20% (w/v) sucrose in HEPES buffer (10 mM, pH 8.0). Resuspended membrane samples were then layered on top of a stepwise gradient containing 3.3 mL 73% (w/v) sucrose at the bottom and 6.7 mL 53% (w/v) sucrose in between. Inner (IM) and outer (OM) membranes were separated by centrifugation at 140,000 × *g* at 4 °C, for 16 hours in a SW40-Ti rotor. The IM resided in the interface between the 53% (w/v) and 20% (w/v) sucrose layers and the OM in the interface between the 53% (w/v) and 73% (w/v) sucrose layers. Both IM and OM samples were removed from the sucrose density interface, diluted with 30 mL HEPES buffer (10 mM, pH 8.0), and pelleted by centrifugation at 75,600 × *g* for 45 min. IM and OM were then resuspended in 1 mL of the same HEPES buffer before lipid and protein extractions.

### Proteomics sample preparation, in-gel digestion and nanoLC-MS analysis

IM and OM samples were carefully dissolved in 100 μL 1X LDS loading buffer (Invitrogen) before loading on a precast Tris-Bis NuPAGE gel (Invitrogen) using 1X MOPS running solution (Invitrogen). SDS-polyacrylamide gel electrophoresis was run for approximately 5 min to purify polypeptides in the polyacrylamide gel by removing contaminants. Polyacrylamide gel bands containing the membrane proteome were excised and digested by trypsin (Roche) proteolysis. The resulting tryptic peptides were extracted using formic acid-acetonitrile (5%:25%, v/v) before resuspension in acetonitrile-trifluoroacetate (2.5%:0.05%, v/v). Tryptic peptides were separated by nano-liquid chromatography (nanoLC) using an Ultimate 3000 LC system with an Acclaim PepMap RSLC C18 reverse phase column (ThermoFisher) at the Proteomics Research Technology Platform (PRTP) at the University of Warwick. MS/MS spectra were collected using an Orbitrap Fusion mass spectrometer (ThermoFisher) in electrospray ionization (ESI) mode. Survey scans of peptides from *m/z* 350 to 1500 were collected for each sample in a 1.5-hr LC-MS run. This resulted in 12 mass spectra (3 biological replicates of IM and OM of WT and the *olsA* mutant) with a total of ~ 7.5 G of MS/MS data.

### MS/MS data search and statistical analyses

Compiled MS/MS raw files were searched against the genome of *Ruegeria pomeroyi* DSS-3 using the MaxQuant software package [17, 18]. Default settings were used and samples were matched between runs. The software package Perseus (v1.6.5.0) was used to determine differentially expressed proteins with a false discovery rate (FDR) of 0.01 [19]. The LFQ (label-free quantitation) intensity of each protein was normalized by dividing the total peptide intensity of each sample by the length of each protein. Peptides were retained for further analyses only if they were consistently found in all three biological replicates in at least one set of the four samples (IM_WT, IM_*olsA*, OM_WT, OM_*olsA*). Missing values were imputed using the default parameters (width, 0.3; down-shift 1.8) and statistical analyses were performed using a two-sample Student’s *t-*test. Principle component analysis (PCA) plots and volcano plots were generated using default settings in the Perseus package.

To analyse the pathways of differentially expressed proteins between the wild-type and the mutant, the sequences of those proteins that were significantly overrepresented (FDR < 0.01) in each sample were annotated using the BlastKOALA program at the KEGG pathway mapping server (https://www.kegg.jp/blastkoala/) using the following settings (taxonomy group-Prokaryotes; KEGG genes databases- family_eukaryotes + genus_prokaryotes).

### Lipidomics analysis of intact polar membrane lipids

Lipid extraction from bacterial cultures was carried out using the modified Folch extraction protocol as described previously [20]. Before lipid extraction from IM and OM samples, they were spiked with d17:1/12:0 sphingosyl phosphoethanolamine (SPE) to a final concentration of 500 nM. Total lipids were then extracted using methanol-chloroform, dried under nitrogen gas and the precipitate resuspended in acetonitrile: 10 mM ammonium acetate at 95:5 (v/v). Lipids were then analysed by LC-MS using a Dionex 3400RS HPLC with a HILIC BEH amide XP column (Waters) coupled with an amaZon SL ion trap MS (Bruker) via electrospray ionisation. After running on the LC-MS, the intensities of each sample peak were calculated using the Bruker QuantAnalysis program and compared against the intensity for SPE. Using the known concentration of SPE, these intensities were then compared to previously created external calibration curves (Supplementary Fig. S2) for each lipid species, including PG and PE. Where no calibration curve was available due to the lack of authentic reference material for a particular lipid species, intensities were compared to curves for lipid species which were structurally similar (e.g., PE for OL, QL, MMPE, DMPE and lysoPE). Using this information, the relative abundance of molar ratio of the lipid species in each membrane fraction could be calculated.

### Minimum inhibitory concentration (MIC) testing

Single colonies of *R. pomeroyi* DSS-3 were cultured in ½ YTSS overnight at 30 °C with shaking (160 rpm). Fresh ½ YTSS was inoculated at a 2% ratio (v/v) and cultured in non-tissue culture treated 24 well plates (Falcon) in the presence of a serial 2-fold dilution of a series of antimicrobial chemicals. Plates were incubated at 30 °C with shaking (150 rpm) and endpoint readings at A_540_ nm were taken at 24 or 48 h in a Fluostar Omega (BMG Labtech) plate reader.

### Membrane permeability and hydrophobicity assays

To measure membrane permeability, crystal violet uptake was monitored using a previously published method [21]. Briefly, cells were grown to an OD_540_ of 0.5–0.6, and 1 mL samples removed for analysis. A solution of crystal violet was prepared in phosphate buffered saline (PBS, pH 7.4) at 10 µg/mL and the OD_590_ measured. Cells were washed by centrifugation at 4500 × *g* for 10 min, removing the supernatant and resuspending the pellet in the same volume of PBS buffer (pH 7.4). The wash step was repeated twice before resuspending the cell pellet in 1 mL PBS + crystal violet (10 µg/mL). Cell suspensions were then incubated at 30 °C for 10 min. After this, cells were harvested by centrifugation at 13,400 × *g* for 15 min, the supernatant removed to a 1 mL cuvette and OD_590_ measured. To measure percentage crystal violet uptake, the following equation was used:

$${{{{{{{\mathrm{Percentage}}}}}}}}\;{{{{{{{\mathrm{uptake}}}}}}}} = \frac{{OD_{590}\;of\;crystal\;violet\;solution - OD_{590}\;of\;supernatant}}{{OD_{590}\;of\;crystal\;violet\;solution}} \times 100$$

*N*-phenyl-1-naphthylamine (NPN) uptake was measured as described previously [22]. Briefly, cultures were grown in ½YTSS media at 30 °C to an OD_540_ of ~ 0.5. Cells were centrifuged for 5 min at 3,000 × *g* and room temperature, before resuspending in half volume of HEPES 5 mM, pH 8.0. Reagents were added in triplicate to a Greiner Bio-One Black 96-well microplate as follows: (1) 200 µL HEPES; (2) 100 µL HEPES + 100 µL bacteria; (3) 150 µL HEPES + 50 µL NPN (40 µM); (4) 50 µL HEPES + 50 µL bacteria + 50 µL NPN (40 µM). All components apart from bacteria were added to the wells in advance, then the bacteria were added immediately before measurement and the fluorescence values began recording within 1 minute. Fluorescence was measured using a BMG Labtech Fluostar Omega microplate reader, with filters set to excitation at 355 nm and emission at 460 nm. All measurements were performed at room temperature. Samples were measured in plate mode every 30 seconds for 10 minutes total.

Cell hydrophobicity was measured using a previously published method [15]. Briefly, cells were grown in ½ YTSS media to an OD_540_ of 0.5–0.6. To a 1.2 mL cell sample, 200 µL hexane were added and samples mixed for 1 min. After waiting 5–10 min to allow phase separation, the lower aqueous phase was removed to a 1 mL cuvette and OD_540_ measured. The percentage adhesion of cells to the organic phase was calculated as follows:

$${{{{{{{\mathrm{Hydrophobicity}}}}}}}} = \frac{{({{{{{{{\mathrm{OD}}}}}}}}_{540}\;{{{{{{{\mathrm{of}}}}}}}}\;{{{{{{{\mathrm{the}}}}}}}}\;{{{{{{{\mathrm{initial}}}}}}}}\;{{{{{{{\mathrm{bacterial}}}}}}}}\;{{{{{{{\mathrm{suspension}}}}}}}} - {{{{{{{\mathrm{OD}}}}}}}}_{540}\;{{{{{{{\mathrm{of}}}}}}}}\;{{{{{{{\mathrm{the}}}}}}}}\;{{{{{{{\mathrm{aqueous}}}}}}}}\;{{{{{{{\mathrm{phase}}}}}}}})}}{{{{{{{{{\mathrm{OD}}}}}}}}_{540}\;{{{{{{{\mathrm{of}}}}}}}}\;{{{{{{{\mathrm{the}}}}}}}}\;{{{{{{{\mathrm{initial}}}}}}}}\;{{{{{{{\mathrm{bacterial}}}}}}}}\;{{{{{{{\mathrm{suspension}}}}}}}}}} \times 100$$

### ^14^C-DMSP synthesis and measurement of radioactive and molar concentration

^14^C-labelled dimethylsulfoniopropionate ([1-^14^C]-DMSP) was synthesised from dimethylsulfide and ^14^C-labelled acrylic acid as described elsewhere [23]. To determine radioactive concentration, 1 μL of synthesized ^14^C-DMSP was added to 3 mL scintillation fluid (EcoLume Liquid Scintillation Cocktail, MP Biomedicals) and measured on a liquid scintillation analyzer (Tri-Carb 2800TR, PerkinElmer) after equilibration overnight. The radioactive concentration of ^14^C-DMSP was measured independently three times and the activity was found to be 0.448 kBq μL^−1^. The identity and molar concentration of DMSP was determined by LC-MS according to a previously published method [24]. ^14^C-DMSP stock in methanol:chloroform:water (12:5:1, v/v) was spiked with 200 nM of deuterated glycine betaine (d_11_-GBT, purchased from Cambridge Isotope Laboratories Inc.) as internal standard (ISTD). Samples were separated on a Discovery HS F5 column (Supelco) with guard column (HS F5 Supelguard) (both obtained from Sigma-Aldrich) using a Dionex 3400RS HPLC and masses detected using an amaZon SL ion trap MS (Bruker) via ESI in positive ion mode. Peak areas of DMSP were detected at *m/z* 135 and of ISTD at *m/z* 129 and determined in each sample using Bruker QuantAnalysis software. The peak area ratio of DMSP:ISTD for each sample was compared to a calibration curve (Supplementary Fig. S3). Therefore, triplicate DMSP standards (dimethylpropiothetin hydrochloride, Supelco via Merck) covering a concentration range from 0.005–2 μM in methanol:chloroform:water (12:5:1, v/v) were spiked with 200 nM d_11_-GBT, measured and peak areas determined as described above. The ratio of DMSP to ISTD allowed to quantify the molar concentration of DMSP in the ^14^C-DMSP stock, which was 328.8 ± 15.2 mM.

### Uptake assays of ^14^C-labelled compounds

A single colony of the WT and the *olsA* mutant was inoculated into 5 mL marine broth 2216 (MB) medium, grown for ~ 24 h at 30 °C with shaking (170 rpm). For growth in ½ YTSS, cells were pelleted by centrifugation (1000 x *g*, 3 min), washed twice in ½ YTSS and inoculated into 50 mL ½ YTSS medium at a 1% (v/v) ratio. Cells were grown for 4 h at 30 °C with shaking (170 rpm) until an OD_540_ of ~0.2 was reached and used for the choline uptake assay. For growth in MAMS medium with 2 mM Pi, cells were pelleted by centrifugation (1000 x *g*, 3 min), washed twice in MAMS medium with 2 mM Pi and inoculated at a 1% ratio (v/v) into 50 mL MAMS with 2 mM Pi. Cells were grown for 5 days at 30 °C with shaking, then pelleted by centrifugation (1000 x *g*, 3 min), washed once in MAMS with 2 mM Pi and inoculated at a 1% ratio (v/v) into 50 mL fresh MAMS with 2 mM Pi. For growth in MAMS medium with 0.5 mM Pi, cells were pelleted, washed and pre-grown in MAMS with 2 mM Pi as described above. Then cells were pelleted by centrifugation (2000 x *g*, 3 min) and washed once in MAMS medium with 0.5 mM Pi. WT cells were inoculated at a 1% ratio and *olsA* mutant cells at a 10% ratio (v/v) into 50 mL fresh MAMS with 0.5 mM Pi. Cultures were grown until an OD_540_ of 0.2–0.4 was reached, then used for the choline uptake assay.

For uptake assays using [methyl-^14^C] choline (55.2 mCi mmol^−1^, Perkin Elmer), cells were diluted 1:1 (v/v) in the same fresh medium and 8 concentrations (0.5–25 µM) of choline (^14^C:^12^C at 1:1000, v/v) were added to 5 mL culture from three biological replicates. Cells were incubated at 30 °C with shaking (130 rpm) for 5 min, harvested by filtration onto 0.2 µm pore size Supor filters (∅ 25 mm diameter, Pall Corporation) and filters washed 3x with 1 mL of the corresponding medium. Filters were transferred into 6 mL scintillation vials, covered with 3 mL scintillation fluid (EcoLume Liquid Scintillation Cocktail, MP Biomedicals) and measured on a liquid scintillation analyser (Tri-Carb 2800TR, PerkinElmer) after equilibration overnight. For background correction, cells were fixed in a final concentration of 2% (v/v) paraformaldehyde solution for a minimum of 15 min at 4 °C in the dark prior to radioisotope addition. Maximum uptake velocity (V_max_) and Michaelis constant (K_m_) were calculated based on Lineweaver-Burke transformation.

For DMSP uptake assays, the WT and the *olsA* mutant were grown for ~24 h at 30 °C with shaking (170 rpm) before inoculating at a 1% v/v ratio into 50 mL fresh marine broth. Cells were grown until an OD_540_ of ~0.5–0.8 was reached before the DMSP uptake assay was carried out. The relatively low radiochemical concentration of the ^14^C-DMSP stock (0.448 kBq μL^−1^, 329 mM) made it impractical to use less than 50 µM DMSP stock for the uptake kinetic assays since the radioactivity would be below the detection limit of the scintillation analyser (Tri-Carb 2800TR, PerkinElmer). Therefore, three high DMSP concentrations (50, 150, and 300 µM) were added to 3 mL of undiluted culture from three biological replicates. Cells were incubated and processed as described above and counts of ^14^C-DMSP for WT and *olsA* mutant cultures measured on the scintillation counter.

To normalise uptake rates, bacterial cell counts were determined by flow cytometry as described previously [25]. Subsamples of 1 mL from each culture were fixed with glutaraldehyde (Electron microscopy grade, BDH) (0.5% (v/v) final concentration) for 30 min at 4 °C, snap frozen in liquid nitrogen and stored at −80 °C. Samples were melted for 5 min at 37 °C and stained with SYBR gold (Invitrogen) (final concentration 10^−4^ of commercial stock) for 10 min at 60 °C in the dark. Samples were diluted between 10- to 50-fold (choline) and 100- to 200-fold (DMSP) in sterile TE buffer (10 mM Tris-HCl, 1 mM EDTA, pH 8.0). Three analytical replicates of each sample were counted on a CytoFLEX flow cytometer (Beckman Coulter) equipped with a 50 mW 488 nm solid-state diode laser and standard filters at a flow rate of 30 µL min^‑1^ for 60 s with the discriminator set to 525 nm (green fluorescence).

### Bacteriophage adsorption assays

To determine the impact of the *olsA* mutant on the interaction of the bacterium with bacteriophages, the rate of adsorption (k) of bacteriophage to the *R*. *pomeroyi* DSS-3 WT and the *olsA* mutant was compared. Bacteriophage DSS3Phi2 was used in this experiment, the first known bacteriophage infecting *R. pomeroyi* DSS-3 [26]. Briefly, culture flasks containing 9 mL bacterial cells at an OD_540_ of 0.5 were kept in a 30 °C water bath, with another flask containing 9 mL ½ YTSS media as a control. After allowing the temperature of the flasks to equilibrate, DSS3Phi2 was added to the flasks at an MOI of 0.1, at which point a timer was started. At time points of 0, 2, 5, 10, 20, and 30 min post-infection, 100 μL bacteria from each flask was removed and added to an Eppendorf tube containing 890 μL ½ YTSS media and 10 μL chloroform, and kept on ice. After the final time point was taken, all samples were centrifuged for 5 minutes at 2000 × *g* and 4 °C to pellet the bacterial cells and the adsorbed phage. The supernatant was then removed into a fresh Eppendorf tube and kept at 4 °C. To determine the phage titre, 100 µL supernatant was removed and serially diluted in Eppendorf tubes containing 900 µL ½ YTSS media down to 10^−9^. 900 µL was removed from the 10^−4^ to 10^−9^ dilutions and added to a 6-well plate. To each well, 1 mL host culture (wild type *R. pomeroyi* DSS-3) at an OD_540_ of approximately 0.3 was added, as well as 4 mL soft ½ YTSS agar (0.8% w/v). Plates were allowed to dry before being incubated overnight at 30 °C. The next day, wells containing 10–100 plaques were counted by eye and used to calculate initial phage titre. The phage adsorption constant was calculated using the following equation ln(P/P_0_) = - kNt where P is the free phage titre after adsorption, P_0_ is the total bacteriophage titre, k is the adsorption constant (ml/ min), N is the bacterial density (cells/ml) and t is time (min) [27].

## Results

### Phospholipids replace aminolipids in the *olsA* mutant in both the IM and OM

**Fig. 1:**
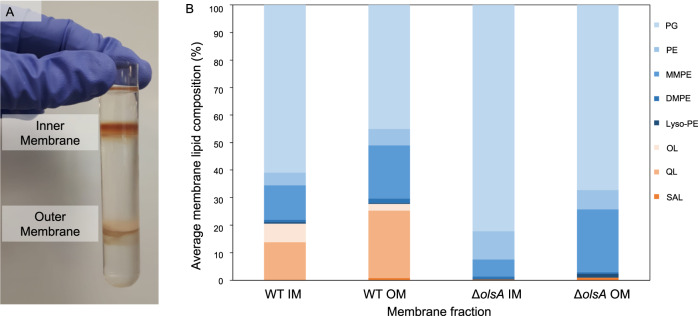
Separation of inner and outer membranes in wild type (WT) *Ruegeria pomeroyi* DSS-3 and the *olsA* mutant cultivated in ½ YTSS medium. **A** Sucrose density gradient separation of the membranes. **B** Lipidomic analyses of inner and outer membrane lipids in the WT and *olsA* mutant. PG phosphatidylglycerol, PE phosphatidylethanolamine, MMPE monomethyl-phosphatidylethanolamine, DMPE dimethyl-phosphatidylethanolamine, lysoPE lyso-phosphatidylethanolamine, OL ornithine-containing aminolipid, QL glutamine-containing aminolipid, SAL sulfonate-containing aminolipid.

To determine the location of QL and OL, we used a stepwise sucrose density gradient to separate the inner membrane (IM) and outer membrane (OM) of the *Ruegeria pomeroyi* DSS-3 WT and *olsA* mutant cultivated in ½ YTSS medium. The IM was consistently found in the interface between 20–53% (w/v) sucrose whereas the OM was at the interface between the 53–73% (w/v) sucrose steps (Fig. 1A). To determine the location of aminolipids in the membrane, intact membrane lipids were extracted from both the IM and OM of the WT strain and analysed by liquid chromatography mass spectrometry (LC-MS). In addition to the commonly seen glycerophospholipids, phosphatidylglycerol (PG) and phosphatidylethanolamine (PE), this bacterium also produced several less abundant phospholipids, including monomethyl-PE (MMPE), dimethyl-PE (DMPE) and lyso-PE, as well as the aminolipids OL and QL (Fig. 1B). Although the aminolipids QL and OL were found in both the IM and OM, QL was more abundant in the OM than in the IM.

To determine the impact of an *olsA* deletion on the membrane lipid profile, we compared the lipid profiles between the WT strain and the mutant (Fig. 1B). All phospholipids were found in the mutant but the aminolipids OL and QL were both absent in the *olsA* mutant due to the essential nature of *olsA* in the final step of QL/OL biosynthesis [4]. PG is by far the most abundant lipid in this bacterium. In the IM, PG accounted for ~60% in the WT and ~80% in the mutant. Similarly, in the OM, PG increased from ~45% in the WT to ~65% in the mutant. Collectively, the lipidomics data suggests that the lack of OL/QL aminolipids in both the IM and OM in the *olsA* mutant is primarily compensated for by a concomitant increase in the phospholipid PG.

### An inability to synthesise aminolipids causes significant changes in membrane properties and a major growth defect

**Fig. 2:**
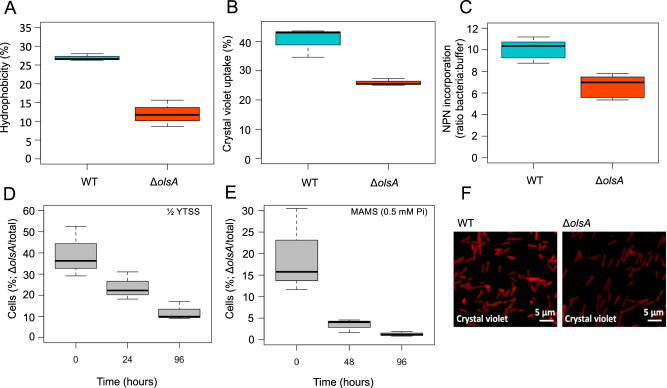
The aminolipid-deficient mutant *olsA* has a growth defect and is uncompetitive compared to the wild type (WT). Mutation of *olsA* cultivated in the ½ YTSS medium caused significant changes in membrane properties, including hydrophobicity (**A**), membrane permeability using crystal violent uptake assay (panels **B** and **F**) and *N*-phenyl-1-naphthylamine (NPN) uptake assay (panel **C**). The *olsA* mutant is less competitive and outcompeted (panels **D** and **E**) by the WT in co-culture in nutrient rich broth medium (1/2 YTSS) and a defined synthetic medium (MAMS, 0.5 mM Pi, glucose 10 mM), respectively.

We determined whether the inability to synthesise aminolipids caused an alteration in membrane properties. Deletion of the aminolipids OL/QL significantly altered membrane hydrophobicity (Fig. 2A) although it remains to be established whether this is due to the direct loss of aminolipids in the OM or a knock-on consequence of the alteration in the OM proteome (see below). The *olsA* mutant displayed significantly reduced permeability as determined by uptake assays using crystal violet (which penetrates the OM but not cytoplasmic IM [28],), *N*-phenyl-1-naphthylamine (NPN) which measures OM permeability (Fig. 2B, C, F) and Eosin Y (Supplementary Fig. S1). We hypothesized such a dramatic change in membrane properties may therefore have unexpected consequences on the growth of the mutant. Thus, we compared the growth rates of the WT and *olsA* mutant in two different types of media, a nutrient rich medium (½ YTSS) and a defined synthetic medium (MAMS). In ½ YTSS medium, although the growth rate of the two strains was comparable when they were grown independently (Table 1), the WT strain clearly outcompeted the *olsA* mutant after 24 h in co-culture (Fig. 2D). By 48 h, the WT was ~9 times more abundant than the mutant. The competitiveness of the WT strain was even more pronounced in defined synthetic MAMS medium. Thus, the growth rate of the mutant was only half of that of the WT in MAMS containing 2 mM Pi and it struggled to grow at 0.5 mM and 0.1 mM Pi in MAMS medium (Table 1). As expected, in co-culture with the WT the mutant was clearly outcompeted after 48 h, by which time the WT was ~20 times more abundant than the mutant (Fig. 2E). These results, therefore, suggest that the inability to synthesise the QL/OL aminolipids represents a major disadvantage for the competitiveness of this bacterium.

**Table 1:** Growth parameters of the wild type and the *olsA* mutant when cultivated separately.

Medium	Growth rate (h^−1^)	
	Wild type	*olsA* mutant
½ YTSS	0.370 ± 0.001	0.387 ± 0.001
MAMS – 2 mM Pi	0.136 ± 0.008	0.072 ± 0.009
MAMS – 0.5 mM Pi	0.146 ± 0.005	0.008 ± 0.058
MAMS – 0.1 mM Pi	0.123 ± 0.010	N. A.

N.A. not available as the mutant was unable to grow. Values are means ± S.D.

### Replacement of aminolipids by PG dramatically changes the membrane proteome

**Fig. 3:**
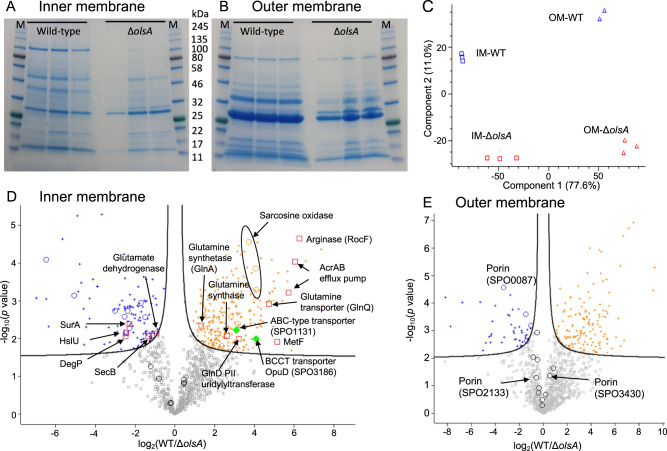
Proteomics analysis of the inner and outer membranes of the wild type (WT) and *olsA* mutant of *Ruegeria pomeroyi* DSS-3 cultivated in the ½ YTSS medium. SDS-PAGE of inner membrane proteins (**A**) and outer membrane proteins (**B**) from the WT and *olsA* mutant. M, Protein molecular weight marker. Membrane separation was carried out in three biological replicates. **C** Principal component analysis (PCA) showing that membrane type is the key driver for separation. **D** Differentially expressed proteins in the inner membrane (IM) between the WT and the *olsA* mutant, FDR cut-off 0.01. **E** Differentially expressed proteins in the outer membrane (OM) between the WT and the *olsA* mutant, FDR cut-off 0.01. Proteins that are upregulated in the WT are shown in orange and those that are downregulated in the WT are shown in blue. Circles represent the most abundant proteins that are >1% of the proteome (Table 2). Red squares indicate proteins that are discussed in the text and green diamonds indicate the two membrane transporters that are likely involved in betaine/choline/carnitine/DMSP transport.

To gain mechanistic insights into the competitive advantage of the WT strain over the *olsA* mutant, we analysed IM and OM protein profiles using comparative proteomics (see Fig. 3A, B). Principle component analysis (PCA) showed a clear separation of the IM and OM samples between the WT and *olsA* mutant strains, with membrane type explaining 77% of the difference (Fig. 3C). Deletion of the *olsA* gene explained only 11% of the total difference according to PCA analysis, suggesting that the difference in proteome is primarily driven by membrane type.

In the IM samples, a total of 1121 proteins were identified, of which 387 were significantly different between the WT and *olsA* mutant (Fig. 3D). The IM is dominated by 24 proteins which account for >55% of the total IM proteome, 13 of which are significantly different between the WT and the mutant (Table 2). This suggests that the loss of aminolipids causes a dramatic change in the IM proteome. In the OM samples, a total of 188 proteins were differentially expressed in the mutant compared to that of the WT (Fig. 3E). The OM is dominated by 15 proteins that account for ~80% of the total OM proteome (Table 2), in particular three uncharacterised membrane porins, one of which (SPO3430) alone accounted for >30% of the total OM proteome.

**Table 2:** Proteomics identification of major membrane proteins in *Ruegeria pomeroyi* DSS-3 and the *olsA* mutant^1^.

Sign.^2^	*p*-value	Diff.^3^	Gene name (locus tag)	Annotation	length	Relative abundance (%)	
IM						*olsA*	WT
	0.517	−0.233	SPO2231	Uncharacterized protein, bactofilin BacA	171	12.7	10.8
	0.055	−1.214	SPO3430	Outer membrane porin	337	11.5	5.0
	0.155	0.433	SPO3162 (*atpD*)	ATP synthase subunit beta	474	6.1	8.3
+	0.001	−1.722	SPO3576	OmpA domain protein	221	3.7	1.1
+	0.000	−6.485	SPO2567	Uncharacterized protein	149	3.6	0.0
+	0.001	−5.076	SPO0087	Uncharacterized protein	351	3.5	0.1
	0.123	0.448	SPO3164 (*atpA*)	ATP synthase subunit alpha	512	3.2	4.4
+	0.002	−2.394	SPO2133	Outer membrane protein	178	2.1	0.4
+	0.002	−3.007	SPO2289	Uncharacterized protein	420	1.6	0.2
	0.115	−0.807	SPO2290	Uncharacterized protein	368	1.5	0.9
+	0.003	−2.530	SPO1670 (*bamA*)	Outer membrane protein assembly factor BamA	787	1.4	0.2
	0.509	−0.191	SPO1330 (*hflC*)	Protein HflC	291	1.3	1.2
+	0.005	−2.285	SPO3617	Peptidoglycan-binding protein	385	1.3	0.3
+	0.003	−1.509	SPO0398	Uncharacterized protein	391	1.1	0.4
	0.273	0.261	SPO3165 (*atpH*)	ATP synthase subunit delta	186	0.9	1.0
	0.278	0.513	SPO1329	Protein HflK	383	0.8	1.1
	0.146	0.456	SPO3163 (*atpG*)	ATP synthase gamma chain	291	0.8	1.0
+	0.004	3.175	SPO1293 (*phaP*)	Phasin, PhaP	148	0.5	4.1
+	0.001	4.076	SPO2345	Sarcosine oxidase, alpha subunit	1010	0.3	5.4
+	0.003	2.804	SPO0316	Electrotransfer ubiquinone oxidoreductase protein	548	0.3	1.8
+	0.000	4.054	SPO2348	Sarcosine oxidase, beta subunit family	414	0.2	3.9
+	0.015	2.343	SPO3772	NADH ubiquinone oxidoreductase	327	0.2	1.0
+	0.000	3.709	SPO2344	Sarcosine oxidase, gamma subunit family	188	0.1	1.4
+	0.001	4.956	SPO3365 (*sitB*)	Manganese ABC transporter, ATP-binding protein	267	0.0	1.0
**OM**						***olsA***	**WT**
	0.044	0.537	SPO3430	Outer membrane porin	337	33.7	48.8
	0.052	−0.596	SPO2133	Outer membrane porin	178	7.4	4.9
+	0.000	−3.307	SPO0087	Outer membrane porin	351	7.0	0.7
	0.001	−0.531	SPO3576	OmpA domain protein	221	6.9	4.8
	0.023	0.853	SPO2290	Uncharacterized protein	368	3.9	7.0
+	0.000	−1.442	SPO2289	Uncharacterized protein	420	3.7	1.3
	0.009	−0.808	SPO1670	Outer membrane protein assembly factor BamA	787	3.1	1.8
	0.012	−0.480	SPO3617	Peptidoglycan-binding protein, putative	385	2.8	2.0
+	0.002	−1.373	SPO0504	Uncharacterized protein	207	2.2	0.8
	0.210	0.211	SPO1240 (*tolC*)	Type I secretion outer membrane protein, TolC	472	1.9	2.2
	0.176	−0.379	SPO2077	Twin-arginine signal sequence domain protein	190	1.5	1.2
	0.527	−0.076	SPO2329	PQQ enzyme repeat family protein	425	1.4	1.3
	0.323	−0.097	SPO3431	Uncharacterized protein	168	1.3	1.2
+	0.001	−1.013	SPO0076	Uncharacterized protein, putative lipoprotein	172	1.1	0.5
	0.119	−0.341	SPO0398	Uncharacterized protein	391	1.0	0.8

1. Only proteins >1% in either the wild-type or the *olsA* mutant are shown in the Table.

2. *p* < 0.01 was considered significant; *IM* inner membrane, *OM* outer membrane.

3. Difference values are log2(fold change), showing the comparison of *olsA* mutant against the wild type. Positive values are proteins that are more abundant in the wild type and negative values represent the proteins that are more abundant in the *olsA* mutant. Protein length in amino acids.

To better understand the impact of aminolipid deficiency on membrane protein profiles and associated cellular processes, we mapped differentially expressed proteins from both the IM and OM using the KEGG pathway mapper with KEGG orthology (KO) classifications. Noticeably, out of 32 identified KO categories, proteins from 2 KO categories were significantly affected by aminolipid deficiency, namely metabolic enzymes (KO01000) and transporters (KO02000) (Table S1). In the KO01000 category, the *olsA* mutant downregulated proteins required for the synthesis/uptake of glutamine and ornithine in response to the lack of glutamine-/ornithine-containing aminolipids in the membrane. The arginase (SPO2119) responsible for ornithine production and the glutamine ABC transporter (SPO3043) are both downregulated in the mutant (Supplementary Fig. S4), suggesting that aminolipid biosynthesis represents a major metabolic drain for nitrogen in this bacterium. This is further supported by over-representation of the glutamine synthetase–glutamate synthase (GS-GOGAT, SPO0765-SPO3272) and the PII protein (SPO0397) in the WT and the glutamate dehydrogenase (GDH, SPO1743) in the *olsA* mutant, respectively. The PII protein, together with the ATP-dependent GS-GOGAT enzyme complex, are important for efficient assimilation of low concentrations of ammonium whereas glutamate dehydrogenase generally has a lower affinity for ammonium.

Expression of an AcrAB efflux pump homolog (SPO1397-1398) is also significantly downregulated in the *olsA* mutant (Fig. 3D, Table S2). We therefore investigated whether this leads to an increase in antibiotic susceptibility given the role of this class of efflux pumps in exporting antibiotics and other toxic molecules from the cell. Susceptibility was increased towards a number of antibiotics from different classes (Supplementary Fig. S5), suggesting this decrease in resistance is caused by the decrease in efflux pump expression due to alteration in the mutant’s lipid profile. This phenotype did not occur for all antibiotics tested, given the broad-spectrum but not universal activity of AcrAB efflux pumps.

### Replacement of aminolipids by PG affects the abundance of membrane transporters and transporter efficiency

**Fig. 4:**
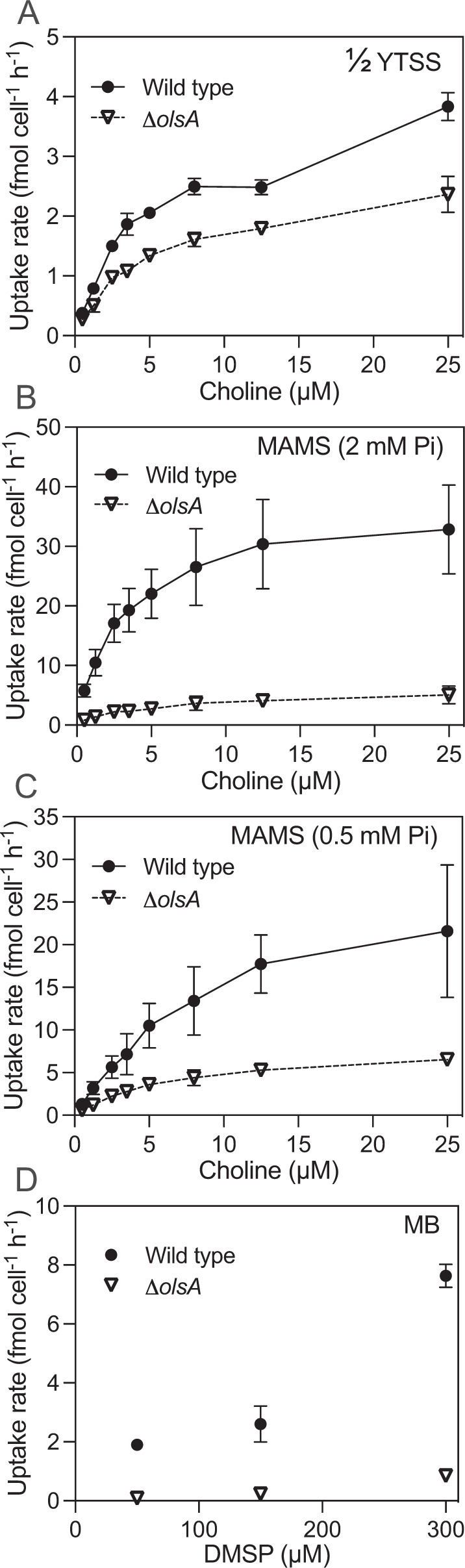
The aminolipid-deficient *olsA* mutant has a defect in membrane transporter activity for choline and dimethylsulfoniopropionate (DMSP) uptake. ^14^C-choline uptake in the wild type (WT) and *olsA* mutant in **A** ½ YTSS medium, **B** MAMS medium with 2 mM Pi and **C** MAMS medium with 0.5 mM Pi. **D** ^14^C-DMSP uptake in the WT and *olsA* mutant at three defined concentrations. Cells were cultivated in the marine broth (MB) medium to induce DMSP uptake. The data presented is the mean of biological triplicates except for the *olsA* mutant in MAMS medium with 0.5 mM Pi, where *n* = 4. Error bars denote standard deviation.

The aforementioned KEGG pathway analysis uncovered dramatic changes in the abundance of membrane transporters in the aminolipids-deficient mutant. In the IM fraction, 48 transporter proteins were more abundant in the WT than in the *olsA* mutant. Indeed, many of those highly represented IM proteins in the WT strain are membrane nutrient transporters, e.g., for amino acids (SPO3706, SPO0823, SPO1849), polyamines (PotA, PotF, PotG), organic phosphorus (UgpB) and inorganic metals (Mg^2+^) (Supplementary Table S2). In particular, the comparative proteomics data suggested that choline metabolism was potentially severely affected in the mutant with two membrane transporters likely involved in quaternary amine transport significantly over-represented in the WT compared to the *olsA* mutant strain, i.e., an organic solute transporter of the betaine-choline-carnitine (BCCT) family (SPO3186) and an ABC-type transporter of the betaine-choline-carnitine family (SPO1131) (Fig. 3D). Choline is an important nutrient for marine roseobacters and the ability to use choline as a carbon/nitrogen source is widespread in roseobacter clade bacteria [16]. Choline degradation leads to the formation of sarcosine and proteins involved in sarcosine oxidation are also significantly over-represented in the WT inner membrane fraction (Fig. 3D). This is further supported by increased expression of MetF, a key enzyme in the one-carbon oxidation pathway of quaternary amines (Fig. 3D, 14). We thus tested choline transport activity in the WT and *olsA* mutant using ^14^C-choline at a range of concentrations (0.5–25 µM). The uptake of choline by WT *Ruegeria pomeroyi* DSS-3 followed Michaelis-Menten kinetics. In ½ YTSS medium, the maximum velocity for choline transport (V_max_) was significantly reduced in the *olsA* mutant (Fig. 4A). This deficiency in choline transport was even more pronounced when cells were grown in defined MAMS medium (Fig. 4B, C). Here, the V_max_ of the WT was ~8 times higher than that of the *olsA* mutant (Table 3). Similarly, we also tested for DMSP transporter activity of the WT and *olsA* mutant by applying three different concentrations of ^14^C-DMSP (50, 150 and 300 µM) using cultures grown in marine broth medium. Uptake in the *olsA* mutant was strongly reduced in comparison to the WT being ~21 times higher in the WT at 50 µM DMSP. At 150 and 300 µM DMSP uptake in the WT was still ~11 and ~9 times higher than in the *olsA* mutant (Fig. 4D). Our data suggests that the inability to produce aminolipids indeed compromises membrane transporter activity for essential nutrients like choline and DMSP in this bacterium. Taken together, the reduced efficiency of an array of membrane transporters in the *olsA* mutant due to the loss of QL/OL aminolipids likely explains the lack of competitiveness of the mutant strain against the WT in co-culture (Fig. 2D, E).

**Table 3:** Uptake kinetics for ^14^C-choline by the wild type and the *olsA* mutant. Values are means ± S.D.

Medium	K_m_ (µM)		V_max_ (fmol cell^−1^ min^−1^)	
	Wild type	*olsA* mutant	Wild type	*olsA* mutant
½ YTSS	5.22 ± 1.38	3.75 ± 0.13	4.81 ± 0.65	2.61 ± 0.17
MAMS – 2 mM Pi	2.42 ± 0.17	2.05 ± 0.26	33.44 ± 7.41	4.12 ± 1.07
MAMS – 0.5 mM Pi	13.25 ± 2.29	5.37 ± 1.27	35.51 ± 8.00	7.15 ± 1.52

### The aminolipid-deficient mutant is less susceptible to phage attachment

**Fig. 5:**
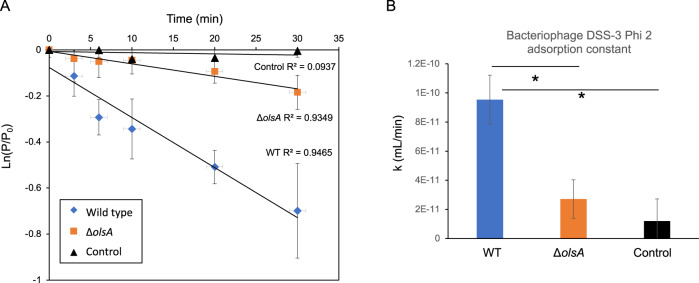
The aminolipid-deficient *olsA* mutant is less susceptible to phage attachment than the wild type (WT). **A** Phage binding kinetics of phage DSS3-Phi2 to WT (blue diamonds), *olsA* mutant (orange squares) and a media-only control (black triangles). **B** Adsorption constant. The data presented is the mean of biological triplicates. Error bars denote standard deviation. Cells were cultivated in the ½ YTSS medium. *p < 0.01, t-test.

We have previously shown that PlcP-mediated membrane lipid remodelling imposes trade-offs for the survival of *Phaeobacter* sp. MED193 [29]. To further understand the ecological importance of these aminolipids, we compared the ability of phage DSS3-Phi2, the first known bacteriophage to infect *Ruegeria pomeroyi* DSS-3 [26], to bind to the WT and the *olsA* mutant. We hypothesised that aminolipids might interact with cell-surface components that could be involved in the phage attachment process and therefore mutation of *olsA* may impact phage adsorption. We performed a phage binding assay on the WT, the *olsA* mutant and a media-only control, measuring phage adsorption over a 30-minute period (Fig. 5A). Indeed, our data revealed that this phage is less able to bind to the *olsA* mutant than the WT with the adsorption constant being ~4 fold less for the mutant (Fig. 5B).

## Discussion

We have shown that QL and OL aminolipids are located in both the IM and OM of this Gram-negative diderm bacterium. Our results corroborate studies in *Paracoccus denitrificans* which also showed that OLs are located in the OM [30]. The OM of Gram-negative bacteria represents a major physical barrier for bacteria to communicate with the surrounding environment. Our proteomics analysis showed that the OM of *Ruegeria pomeroyi* DSS-3 was dominated by a single OM porin (SPO3430), which accounted for ~49% of the total OM proteome in the WT and ~34% in the *olsA* mutant although this difference was not statistically significant (Table 2). Membrane porins in marine roseobacter clade bacteria in general are poorly studied [31, 32]. In *Roseobacter denitrificans*, it is estimated that pore-forming homotrimer porins cover as much as ~70% of the OM surface. Moreover, these types of porin are cation-selective, although the exact solutes they transport remains to be established [31].

Our lipidomics analyses showed that the aminolipids OL and QL are major lipids in the membrane of this bacterium and that loss of these lipids is primarily compensated for by the phospholipid PG. It appears that aminolipid production in the WT strain represents a major drain for nitrogen flow. Comparative proteomics showed that the WT strain significantly overproduced transporters for organic nitrogen species and the high-affinity ammonium assimilation pathway involving glutamine synthetase (GS) and glutamate synthase (GOGAT) was switched on. Enzymes responsible for ornithine formation from arginine were also upregulated. In contrast, in the *olsA* mutant the high-affinity GS-GOGAT pathway was down-regulated and the low-affinity glutamate dehydrogenase pathway preferred instead. Such a transition in the ammonium assimilation pathway strongly suggests that the WT strain has a higher demand for nitrogen in order to synthesize sufficient aminolipids to incorporate in the membrane, while benefiting from a lower demand for phosphorus. Our finding has important implications for better understanding the ecophysiology of these cosmopolitan marine bacteria, suggesting that they have optimized their cellular demand in response to phosphorus scarcity in the surface ocean. In this regard, it is worth noting that many marine roseobacters are also capable of remodelling membrane lipids by replacing phospholipids with sugar-containing phosphorus-free glycolipids [3].

In *R. pomeroyi*, we detected 1121 proteins associated with the IM. The number of IM proteins detected in *R. pomeroyi* is in line with the number of IM proteins in *E. coli* and indeed, the chromosome sizes are comparable (4.6 Mbp vs 4.1 Mbp). It is predicted that *E. coli* has ~1000 integral IM proteins, ~570 peripherally associated with the IM and 24 IM lipoproteins [33]. Abolishing aminolipid biosynthesis in the *olsA* mutant caused a dramatic change in membrane protein composition, with the abundance of ~35% of the proteins (387 out of 1121 detected) associated with the IM of *R. pomeroyi* DSS-3 changing significantly, highlighting that specific protein-lipid interactions play an important role in maintaining “optimum” membrane function in this bacterium. When investigating changes in the IM proteome between the WT and the *olsA* mutant, it is striking that ~60 membrane transporter proteins are differentially regulated by these aminolipids. Using ^14^C-labelled substrates, we were able to validate the results predicted from comparative proteomics, directly showing that uptake of both ^14^C-choline and ^14^C-DMSP are indeed hampered by a lack of aminolipids in the membrane. This is likely due to the fact that membrane transporters require specific lipids in order to maintain a favourable confirmation for efficient solute transport. Previous work in *Escherichia coli* has shown that the membrane transporter for betaine, BetP, requires specific interactions with the anionic lipid PG in order to maintain a functional homotrimer [34]. Such a specific interaction between membrane proteins and membrane lipids likely affects the fitness of the bacterium in terms of nutrient acquisition capacity and may help to explain the competitiveness of the WT strain over the aminolipid-deficient mutant (*olsA*). However, a more detailed structure-function characterization of membrane protein interactions with these aminolipids is required to obtain a precise mechanistic understanding of this process, which in turn will lead to a better understanding of the ecophysiological trade-offs we have observed here. Similarly, we were able to validate the prediction that the reduction in AcrAB efflux pump (SPO1397-1398) in the absence of aminolipids led to an increase in sensitivity to a number of antibiotics.

The lack of aminolipids in the *olsA* mutant also resulted in a significant increase in the abundance of several proteins involved in the assembly of OM components, including OM β-barrel proteins (BAM complex), lipopolysaccharides (LPS) transporters and several OM porins. Notably the BAM complex and LPS transporter proteins are significantly overproduced in the *olsA* mutant (Table 2, Supplementary Table S2, S3). Lipidomics analyses suggest the bacterium compensates for the loss of aminolipids in the OM by increasing PG formation. It is plausible that such an aminolipid-to-PG substitution results in an inability to maintain the abundance of the major OM porins (SPO3430, down from 48 to 34% in the mutant). In order to maintain OM integrity, other OM components (i.e., the BAM complex, LPS, other OM porins) are overproduced to compensate for the loss of this major OM porin (i.e., SPO3430). The need to upregulate components of the protein export machinery is also reflected in the overproduction of SecB (SPO3888) in the mutant. Such an emergency rescue strategy due to the loss of aminolipids causing OM trauma is also reflected in the production of OM stress response proteins in the *olsA* mutant (Supplementary Table S3), molecular chaperones for Sec-dependent OM secretion and maturation (e.g., DegP (SPO1625), SurA (SPO2456)) and a stress-induced proteinase HslU (SPO3882) [35–37]. These findings corroborate with the transporter activity assays and competition assays between the WT and *olsA* mutant, suggesting that although the loss of aminolipids can be compensated for by over-representation of PG to some extent, such an aminolipid-to-PG substitution is far from optimal for the bacterium, reducing its competitiveness.

Aminolipids also appear to play a role in the ability of phage DSS3-Phi2 to bind to its host. Our phage binding assay showed that when aminolipids are present, the rate of phage attachment is ~4 times higher than in the *olsA* mutant, indicating a higher risk of phage lysis. This has ecological implications, suggesting a trade-off between bacterial fitness and susceptibility to phage infection. A similar phenomenon was shown previously in a bacteria-protist grazer scenario. Bacteria which undergo PlcP-mediated lipid remodelling in response to low phosphorus were less likely to be captured by grazers but became more susceptible to digestion once captured [29]. Similarly, it appears aminolipid formation, which is independent of the PlcP-mediated lipid remodelling pathway, confers a competitive advantage in the form of nutrient uptake, but exerts trade-offs by rendering the cell more prone to bacteriophage infection. Although the phage receptor for bacteriophage DSS3Phi2 remains to be identified, it is tempting to speculate that these aminolipids may be instrumental for maintaining the abundance and/or correct confirmation of phage receptors in the cell surface of the bacterium. This certainly warrants further investigation. The role of these aminolipids in interactions with protist grazers is less clear since the *olsA* mutant and the WT *Ruegeria pomeroyi* behave indistinguishably when challenged with the ciliate *Uronema marinum* (data not shown). It therefore appears that the bacterium must finetune the production of aminolipids to balance between nutrient uptake ability and lysis by phage.

Taken together, our results demonstrate that aminolipids play an integral role in the ecophysiology of a cosmopolitan marine bacterium. Given that ornithine and glutamine lipids are ubiquitous in marine roseobacter clade bacteria, it is therefore likely that MRC have fine-tuned their membrane lipid composition to maintain their competitiveness in marine microbial ecosystems.

## Supplementary information

Fig S1

Fig S2

Fig S3

Fig S4

Fig S5

Table S1

Table S2

Table S3

## Data Availability

All data is included in the manuscript. Bacterial strains, mutants, and phages are available upon request.
