# Supplementary material for: Aminolipids elicit functional trade-offs between competitiveness and bacteriophage attachment in Ruegeria pomeroyi
Source: ISME J. 2022 Dec 7;17(3):315–25. doi: 10.1038/s41396-022-01346-0 (PMC9938194; doi:10.1038/s41396-022-01346-0)
Supplement: Supplementary file 1 — Fig S1 [file 41396_2022_1346_MOESM1_ESM.docx]

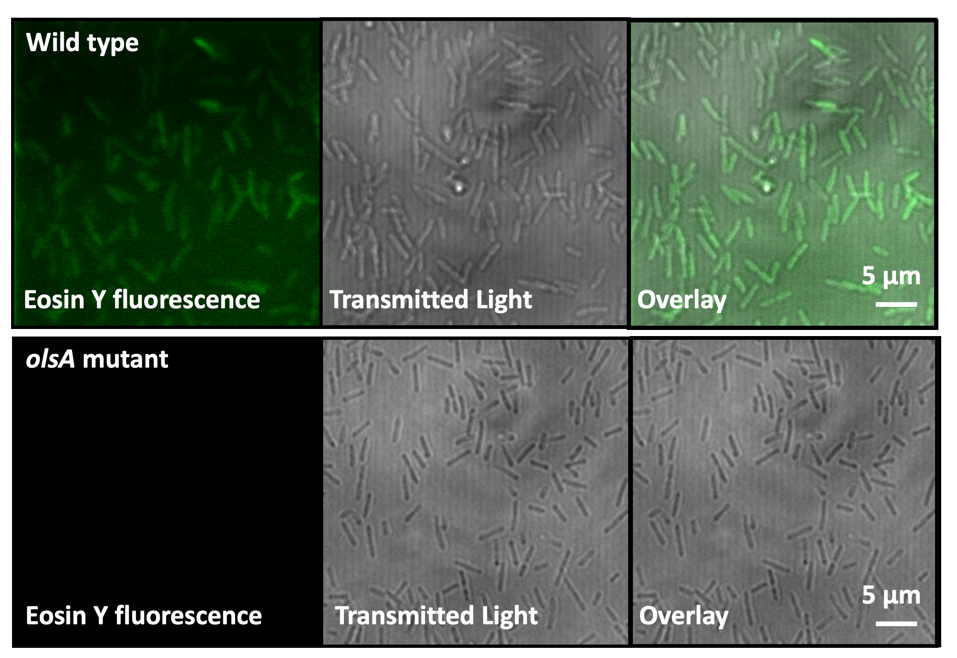


**Supplementary Figure S1** Mutation of *olsA* caused significant changes in membrane staining properties using Eosin Y. While the wild type cells were stained uniformly by Eosin Y, the *olsA* mutant cells were unable to bind to the dye.
