# Supplementary material for: Aminolipids elicit functional trade-offs between competitiveness and bacteriophage attachment in Ruegeria pomeroyi
Source: ISME J. 2022 Dec 7;17(3):315–25. doi: 10.1038/s41396-022-01346-0 (PMC9938194; doi:10.1038/s41396-022-01346-0)
Supplement: Supplementary file 2 — Fig S2 [file 41396_2022_1346_MOESM2_ESM.docx]

**Supplementary Fig S2** Calibration curves for the quantitation of bacterial membrane lipids. SQDG, sulfoquinovosyl diacylglycerol; PE, phosphatidylethanolamine; PG, phosphatidylglycerol, SPE Sphingosyl phosphoethanolamine. (d17:1/12:0)
