# Supplementary material for: Aminolipids elicit functional trade-offs between competitiveness and bacteriophage attachment in Ruegeria pomeroyi
Source: ISME J. 2022 Dec 7;17(3):315–25. doi: 10.1038/s41396-022-01346-0 (PMC9938194; doi:10.1038/s41396-022-01346-0)
Supplement: Supplementary file 3 — Fig S3 [file 41396_2022_1346_MOESM3_ESM.docx]

**Supplementary Figure S3** Calibration curve for the quantification of the molar concentration of dimethylsulfoniopropionate (DMSP) showing a linearity over the range 0.005-2 μM. Standards were run in triplicate and d_11_-glycine betaine (d_11_-GBT) at a concentration of 200 nM was used as an internal standard (ISTD).
