# Supplementary material for: Aminolipids elicit functional trade-offs between competitiveness and bacteriophage attachment in Ruegeria pomeroyi
Source: ISME J. 2022 Dec 7;17(3):315–25. doi: 10.1038/s41396-022-01346-0 (PMC9938194; doi:10.1038/s41396-022-01346-0)
Supplement: Supplementary file 4 — Fig S4 [file 41396_2022_1346_MOESM4_ESM.docx]

**
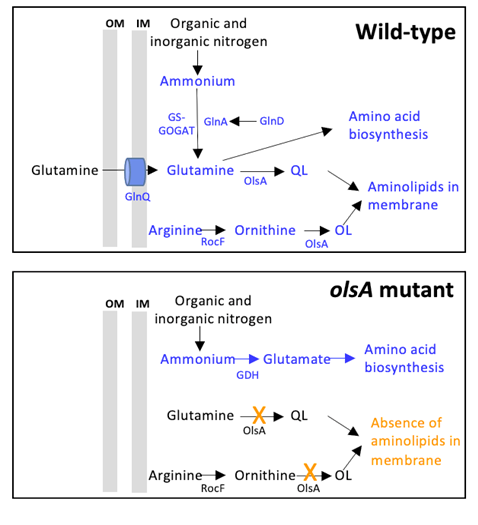
**

**Supplementary Figure S4** A working model of ammonium assimilation and aminolipid biosynthesis between the wild type and the *olsA* mutant. The aminolipid-deficient *olsA* mutant was unable to synthesise glutamine-/ornithine lipids (QL and OL, respectively), which appears to downregulate proteins involved in ammonium assimilation and glutamine uptake. The glutamine synthetase -glutamate synthase (GS-GOGAT) pathway was also downregulated in the *olsA* mutant. GlnA, glutamine synthetase; GlnD, PII uridylyltransferase; GlnQ, glutamine transporter membrane protein; RocF, arginase; GDH, glutamate dehydrogenase.
