# Supplementary material for: Aminolipids elicit functional trade-offs between competitiveness and bacteriophage attachment in Ruegeria pomeroyi
Source: ISME J. 2022 Dec 7;17(3):315–25. doi: 10.1038/s41396-022-01346-0 (PMC9938194; doi:10.1038/s41396-022-01346-0)
Supplement: Supplementary file 5 — Fig S5 [file 41396_2022_1346_MOESM5_ESM.docx]

**
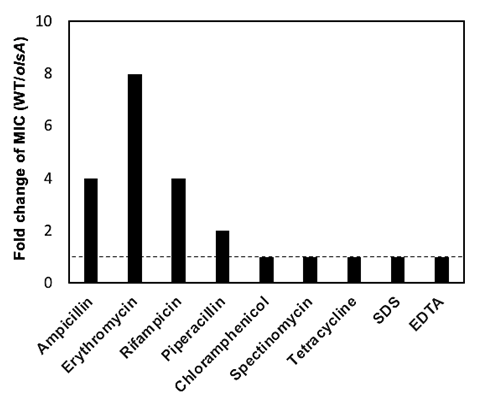
**

**Supplementary Figure S5** Fold change in the minimum inhibitory concentration (MIC) of the WT strain over the *olsA* mutant for selected antimicrobials. The dashed line indicates no change in the MIC between the wild type and the *olsA* mutant. SDS, sodium dodecyl sulfate; EDTA, ethylene-diamine tetra-acetic acid.
